# Hypobaric Intermittent Hypoxia Attenuates Hypoxia-induced Depressor Response

**DOI:** 10.1371/journal.pone.0041656

**Published:** 2012-07-27

**Authors:** Fang Cui, Lu Gao, Fang Yuan, Ze-Fei Dong, Zhao-Nian Zhou, David D. Kline, Yi Zhang, De-Pei Li

**Affiliations:** 1 Department of Physiology, Hebei Medical University, Shijiazhuang, China; 2 Shanghai Institutes for Biological Sciences, Chinese Academy of Sciences, Shanghai, China; 3 Department of Biomedical Sciences and Dalton Cardiovascular Research Center, University of Missouri, Columbia, Missouri, United States of America; 4 Department of Critical Care, The University of Texas, M.D. Anderson Cancer Center, Houston, Texas, United States of America; 5 Hebei Key Laboratory of Medical Biotechnology,Shijiazhuang, China; University of Toronto, Canada

## Abstract

**Background:**

Hypobaric intermittent hypoxia (HIH) produces many favorable effects in the cardiovascular system such as anti-hypertensive effect. In this study, we showed that HIH significantly attenuated a depressor response induced by acute hypoxia.

**Methodology/Principal Findings:**

Sprague-Dawley rats received HIH in a hypobaric chamber simulating an altitude of 5000 m. The artery blood pressure (ABP), heart rate (HR) and renal sympathetic nerve activity (RSNA) were recorded in anesthetized control rats and rats received HIH. The baseline ABP, HR and RSNA were not different between HIH and control rats. Acute hypoxia-induced decrease in ABP was significantly attenuated in HIH rat compared with control rats. However, acute hypoxia-induced increases in HR and RSNA were greater in HIH rat than in control rats. After removal of bilateral ascending depressor nerves, acute hypoxia-induced depressor and sympathoexcitatory responses were comparable in control and HIH rats. Furthermore, acute hypoxia-induced depressor and sympathoexcitatory responses did not differ between control and HIH groups after blocking ATP-dependent K^+^ channels by glibenclamide. The baroreflex function evaluated by intravenous injection of phenylephrine and sodium nitroprusside was markedly augmented in HIH rats compared with control rats. The pressor and sympathoexcitatory responses evoked by intravenous injection of cyanide potassium were also significantly greater in HIH rats than in control rats.

**Conclusions/Significance:**

Our findings suggest that HIH suppresses acute hypoxia-induced depressor response through enhancement of baroreflex and chemoreflex function, which involves activation of ATP-dependent K^+^ channels. This study provides new information and underlying mechanism on the beneficiary effect of HIH on maintaining cardiovascular homeostasis.

## Introduction

Prolonged expose to intermittent hypoxia, which mimics obstructive sleep apnea, is associated with an increase in basal arterial blood pressure and sympathetic outflow in animal and human [1], [2], [3], [4], [5]. Unlike atmospheric intermittent hypoxia, hypobaric intermittent hypoxia (HIH) has been used frequently by athletes to enhance performance through increasing exercise tolerance [6], [7], [8]. In addition, HIH produces many favorable effects in the cardiovascular system such as anti-hypertensive effect [9]. In this regard, HIH decreases arterial pressure in hypertension patients and animals with experimental hypertension [10]. It has been shown that HIH produces anti-hypertensive effect through suppression of sympatho-adrenal and rennin-angiotensin systems [10], [11]. Also, HIH reduces sympathetic activity through an enhancement of baroreflex in patients with sleep apnea [12], [13]. However, HIH does not cause sustained alterations in autonomic control of blood pressure in young athletes and has no effect on the basal homodynamic (including arterial blood pressure) [14], [15].

Acute hypoxia induces a decrease in arterial blood pressure through vasodilatation of various vascular beds and a reduction of the total peripheral blood vessel resistance [16], [17]. Baroreflex is the primary negative feedback mechanism regulating systemic arterial blood pressure and cardiovascular homeostasis. An elevated arterial pressure reflexively causes heart rate and sympathetic outflow to decrease therefore resulting in an arterial pressure decrease; likewise, a decreased blood pressure activates the baroreflex to cause increase in heart rate and sympathetic outflow and result in an arterial pressure increase. The baroreceptors located in the aortic arch and carotid sinus detect a fluctuation and convey this information to nuclei in brainstem such as nucleus tratus solitarius, caudal ventrolateral medulla, and rostral ventrolateral medulla through aortic depressor nerve and carotid sinus nerve [18], [19]. It has been shown that exposure to hypoxia induces a significant increase in parasympathetic drive even after removal of the hypoxic stimulus [20], [21], [22]. This mechanism may be involved in the HIH-induced increases in sensitivity of baroreflex [12], [13]. Acute hypoxia also stimulates chemoreceptors located in the carotid bodies and leads to behavioral, respiratory and autonomic adjustments to restore oxygen levels to physiological range. Activation of chemoreceptor causes an increase in sympathetic drive to the vasculature, and a decrease in heart rate [23], [24]. The increase in sympathetic outflow largely against the depressor response induced by acute hypoxia.

In this study, we determined the anti-depressor action produced by HIH during acute hypoxia and the possible mechanisms. We found that HIH suppresses acute hypoxia-induced depressor response through an increase in sensitivity of baroreflex and chemoreflex, which may involve activation of K_ATP_ channels.

## Results

The body weight in rats received HIH treatment was not significantly different from age-matched control rats during the whole treatment. The body weight of HIH rats and control rats was 246±30 g and 255±25 g in two-week, 279±24 g and 285±24 g in four-week, and 341±34 and 343±21 g in six-week of experiment, respectively. The basal ABP and HR measured were not significantly different between HIH rats and control rats conditions (Table 1). The basal blood pressure recorded through femoral artery in those rats anaesthetized with urethane was 97.2±8.6 mmHg in control, 97.4±20.3 mmHg in CIHH14, 99.8±7.5 mmHg in CIHH28, and 106.7±6.8 mmHg in CIHH42 rats, respectively.

**Table 1:** Effect of HIH on ABP and HR in conscious and anesthetized rats.

	ABP(mmHg)		HR(beat/min)	
	conscious	anesthetized	Conscious	anesthetized
CON	105.3±8.8	97.2±8.6	383.3±41.2	378.1±58.2
HIH14	108.7±6.6	97.4±20.3	395.9±30.1	370.7±26.2
HIH28	115.3±7.4	99.8±7.5	396.1±19.2	368.8±26.4
HIH42	105.9±9.3	106.7±6.8	388.3±19.2	373.4±27.3

All data expressed as mean ± SEM, n = 6 for each group. HIH: chronic intermittent hypobaric hypoxia, ABP: artery blood pressure, HR: heart rate, CON: control, HIH14: HIH for 14 days group, HIH28: HIH for 28 days group, HIH42: HIH for 42 days group.

### The Response of ABP, HR, and RSNA to Acute Hypoxia in Rats Received HIH Treatment

**Figure 1:**
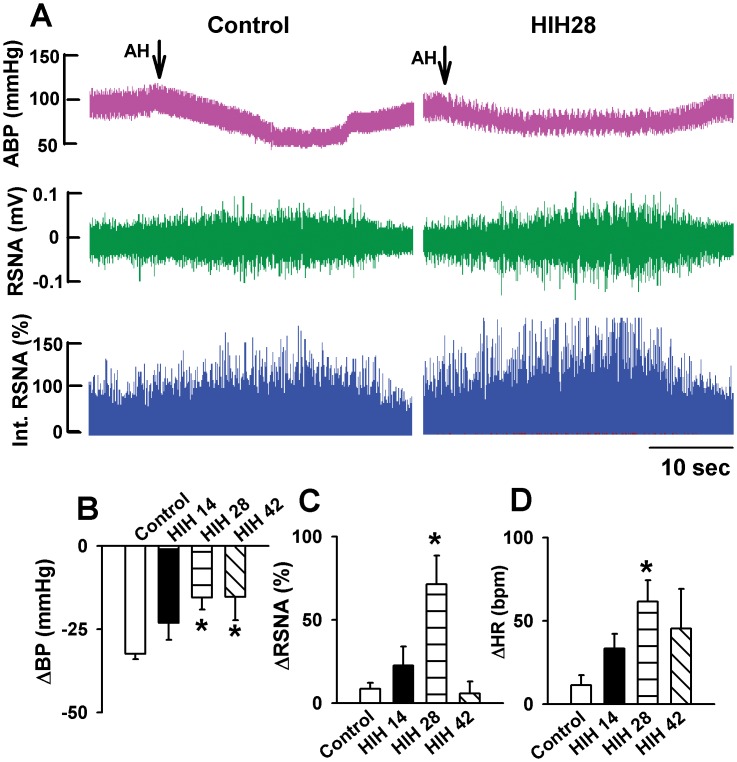
The effect of HIH treatment on acute hypoxia-induced depressor and sympathoexciatory effect in anesthetized rats. A: Original recordings showing the responses of ABP and RSNA to acute hypoxia (O_2_∶10%; N_2_∶90% for 2 min) in one control and one HIH rat. B, C, and D: Summary data showing the acute hypoxia-induced alteration of ABP (B), RSNA (C) and HR (D) in control and HIH-treated rats. The summary measurements were derived from the peak responses of ABP, HR, and RSNA, respectively. AH, acute hypoxia; ABP: artery blood pressure; HR: heart rate; RSNA: renal sympathetic nerve activity. All data expressed as mean ± SEM, n = 7 for each group, *P<0.05 v.s. control group.

During acute hypoxia, ABP significantly decreased 99.0±5.7 to 66.6±5.0 mmHg, while HR and RSNA significantly increased 373.0±63.8 to 384.4±67.5 bpm and 100% to 108.5±3.7% in anesthetized control rats. The decrease in ABP was significantly attenuated, while the increases in HR and RSNA were significantly enhanced in rats received HIH treatment compared with control rats (Figure 1). Furthermore, the decrease of ABP induced by acute hypoxia in rats received HIH for 28 days (n = 7) and 42 days (n = 7) were significantly smaller than that in HIH14 (n = 7) group. Also, the enhancement of HR and RSNA in HIH28 and HIH42 were significantly greater than that in HIH14. After acute hypoxia, ABP, HR and RSNA returned to baseline (Figure 1).

### Role of Ascending Depressor Nerves in Mediating Acute Hypoxia-induced Depressor and Sympathoexcitatory Responses in Control and HIH Rats

**Figure 2:**
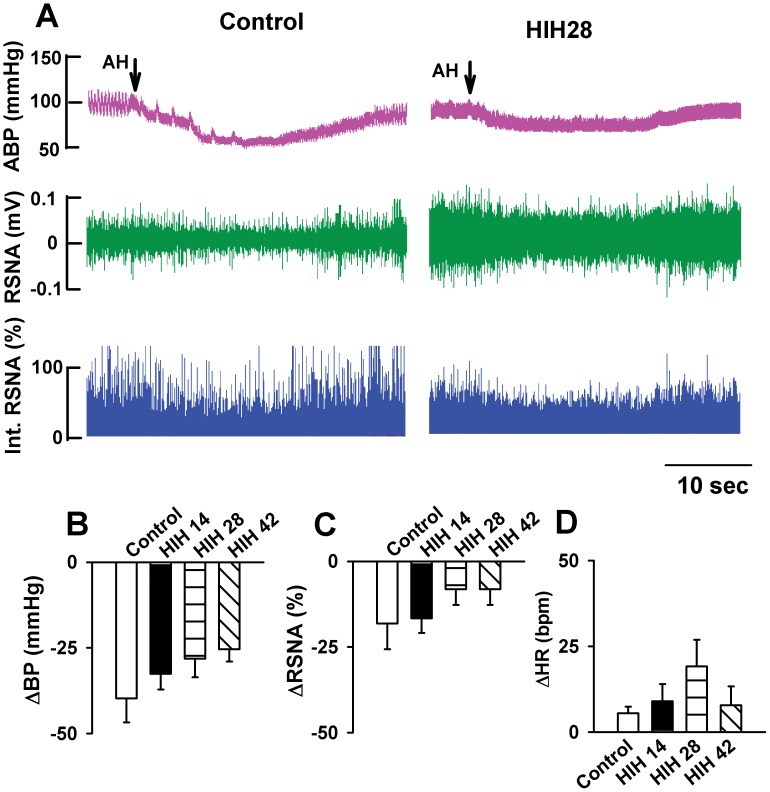
Removal of ascending depressor nerve abolished acute hypoxia-induced increases in RSNA, while enhanced the decrease in ABP in both control and HIH rats. A: Original recordings showing that responses of ABP and RSNA to acute hypoxia after removal of ascending depressor nerve in one control and one HIH rat. B, C, and D: Summary data showing the acute hypoxia-induced alteration of ABP (B), RSNA (C) and HR (D) in control and HIH-treated rats. The summary measurements were derived from the peak responses of ABP, HR, and RSNA, respectively. AH, acute hypoxia; ABP: artery blood pressure; HR: heart rate; RSNA: renal sympathetic nerve activity. All data expressed as mean ± SEM, n = 6 for each group, *P<0.05 v.s. control group.

Removal of bilateral buffer nerves including both aortic depressor nerves and carotid sinus nerves had no effect on basic ABP, HR, and RSNA in control rats. However, denervation of buffer nerve augmented the depressor response induced by acute hypoxia, while attenuated the increases in HR and RSNA induced by acute hypoxia (Figure 2). Compared to control rats, acute hypoxia-induced depressor response was blunted in HIH rats after denervation of buffer nerves (Figure 2). Furthermore, the responses of ABP and RSNA to acute hypoxia in HIH28 and HIH42 were significantly attenuated than that in HIH14 group. There was no significant difference in response of RSNA to acute hypoxia between HIH14 and control groups. The response of HR to acute hypoxia in HIH28 was greater than that in HIH14 and HIH42. However, there were no difference in acute hypoxic responses of HR among control, HIH14 and HIH42. ABP, HR and RSNA returned to baseline levels after acute hypoxia (Figure 2).

### Effect of Glibenclamide and L-NAME on Responses of ABP, HR, and RSNA to Acute Hypoxia

**Figure 3:**
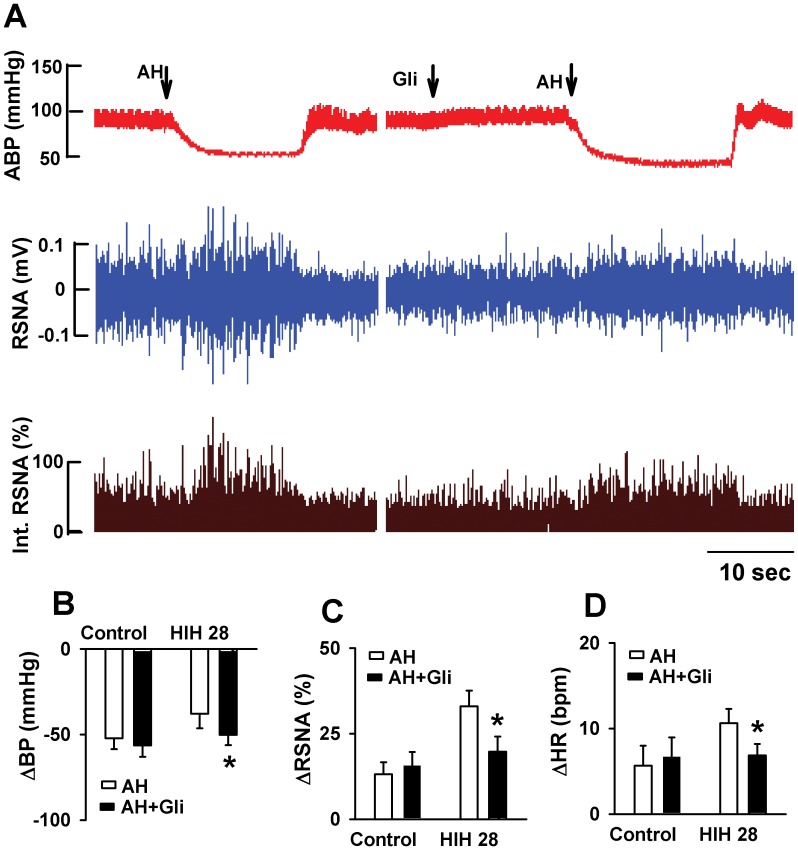
The effect of glibenclamide on the response of ABP, HR, and RSNA to acute hypoxia in control and HIH-treated rats. A: Original recordings showing that responses of ABP and RSNA to acute hypoxia before and after administration of glibenclamide in one control and one HIH rats. B, C, and D: Summary data showing the acute hypoxia-induced alteration of ABP (B), RSNA (C) and HR (D) before and after administration of glibenclamide in control and HIH-treated rats. The summary measurements were derived from the peak responses of ABP, HR, and RSNA, respectively. ABP: artery blood pressure; HR: heart rate; RSNA: renal sympathetic nerve activity; AH: acute hypoxia; Gli: glibenclamide. All data expressed as mean ± SEM, n = 6 for each group, *P<0.05 v.s. control group.

**Figure 4:**
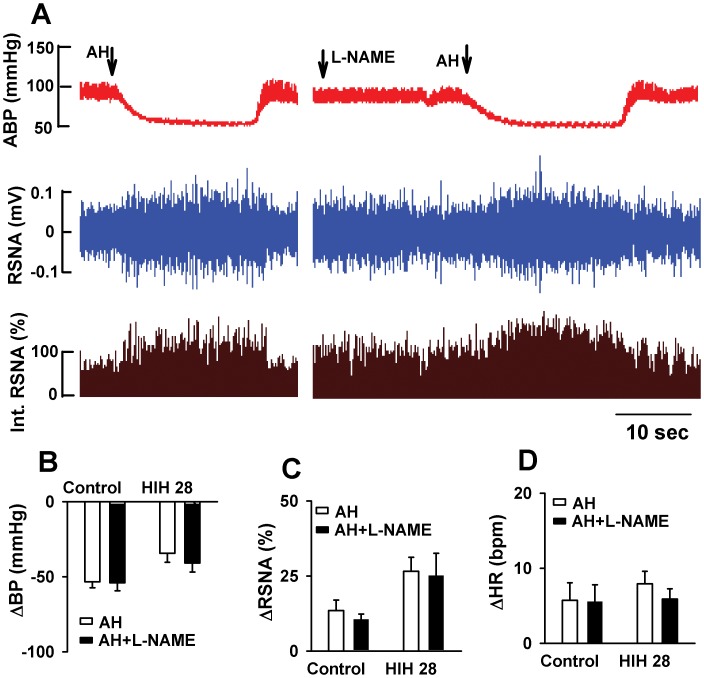
The effect of L-NAME on the response of ABP, HR, and RSNA to acute hypoxia in control and HIH-treated rats. A: Original recordings showing that responses of ABP and RSNA to acute hypoxia before and after administration of L-NAME in one anesthetized control and one HIH rat. B, C, and D: Summary data showing the acute hypoxia-induced alteration of ABP (B), RSNA (C) and HR (D) before and after administration of L-NAME in control and HIH-treated rats. The summary measurements were derived from the peak responses of ABP, HR, and RSNA, respectively. ABP: artery blood pressure; HR: heart rate; RSNA: renal sympathetic nerve activity; AH: acute hypoxia. All data expressed as mean ± SEM, n = 6 for each group.

To determine if ATP-sensitive K^+^ channels (K_ATP_) was involved in the acute hypoxic responses of ABP, HR, and RSNA in HIH rats, glibenclamide (15 mg/kg, 0.1 ml, iv bolus) [25], a specific blocker for K_ATP_ channel, was administered intravenously before acute hypoxia. Glibenclamide alone had no effect on the baseline ABP, HR, and RSNA. However, glibenclamide treatment abolished the response of ABP, HR, and RSNA to acute hypoxia (Figure 3). Because nitric oxide (NO) is critically involved in regulating blood pressure and sympathetic activity [26], then we determine if NO production was involved in the responses of ABP, HR, and RSNA to acute hypoxia. Intravenous injection of endothelial NO synthesase (eNOS) inhibitor L-NAME (5.5 µg/kg, 0.1 ml, iv bolus) alone has not significant effect on the baseline ABP, HR, and RSNA. Furthermore, pretreatment of L-NAME had no effect on the hypoxic change of ABP, HR and RSNA in HIH28 rat (Figure 4).

### Effects of HIH on Baroreflex and Chemoreflex in Anesthetized Rats

**Figure 5:**
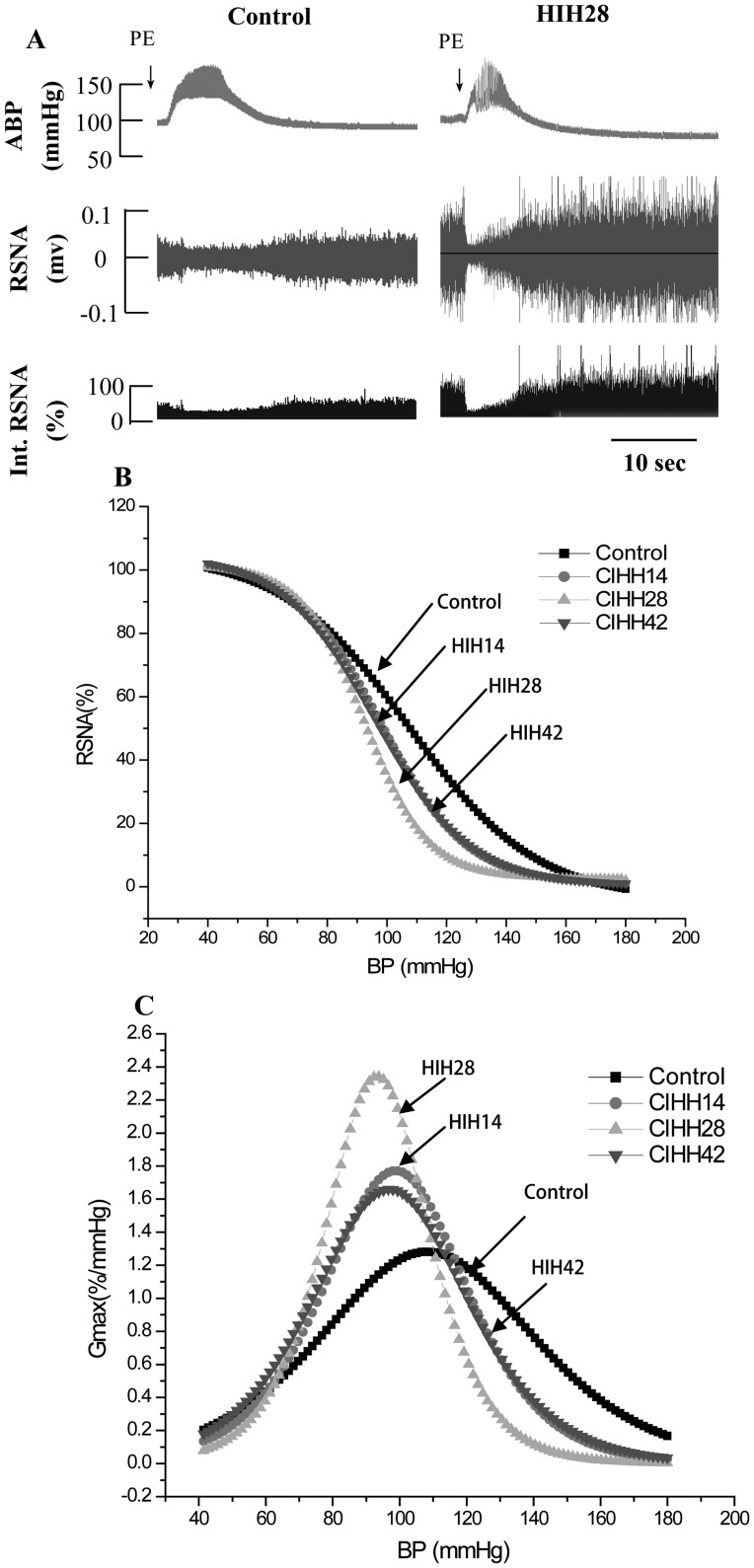
Baroreflex functions in control and HIH-treated rats. A: Original recordings depict the changes in RSNA and HR in response to elevation of ABP induced by intravenous injection of phenylephrine in one control and one HIH rat. B, baroreflex function curves derived from reflex changes in RSNA and HR in 7 control and 8 HIH-treated rats. C, gain of the mean baroreflex curves. The maximum gain values for RSNA in HIH treatment groups (HIH14: −1.7±0.3%·mm/Hg; HIH28: −2.6±0.4%·mm/Hg; and HIH 42: −1.6±0.2%·mm/Hg) were significantly greater than in control rats (−1.3±0.2%·mm/Hg, P<0.05). Int-RSNA: integrated RSNA; PE: phenylephrine.

**Figure 6:**
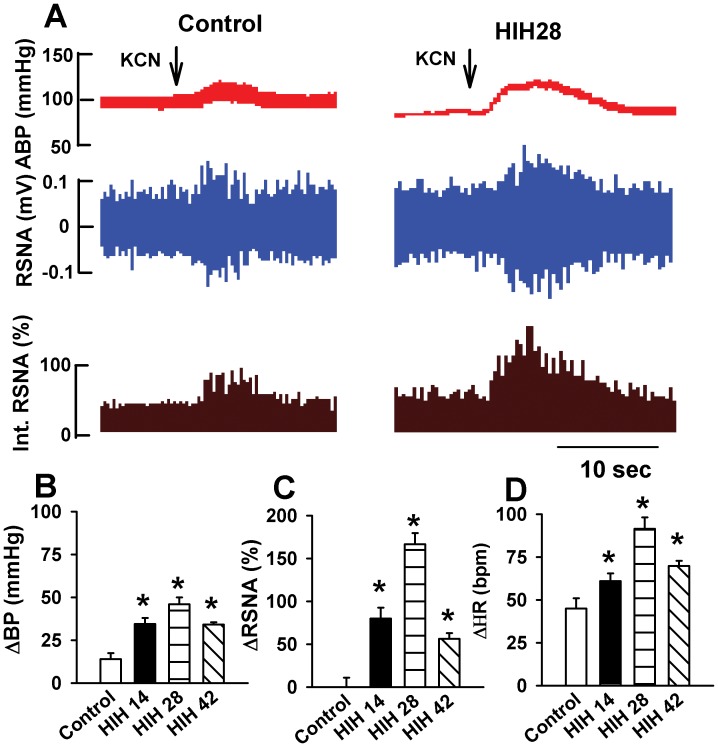
Chemoreflex functions in control and HIH rats. A: Original recordings show the responses of ABP and RSNA intravenous injection of cyanide potassium in one control and one HIH rat. B, C, and D: Summary data showing the effect of intravenous injection of cyanide potassium on ABP (B), RSNA (C), and HR (D) in control and HIH rats. The summary measurements were derived from the peak responses of ABP, HR, and RSNA, respectively. ABP: artery blood pressure; HR: heart rate; RSNA: renal sympathetic nerve activity; KCN: cyanide potassium. All data expressed as mean ± SEM.

To directly assess the baroreflex function, we decreased blood pressure by intravenous injection of sodium nitroprusside (50 µg/kg, 0.1 ml, iv bolus) and raised blood pressure by intravenous injection of phenylephrine (8 µg/kg, 0.1 ml, iv bolus), respectively. The baroreflex curves were shifted to the left in HIH groups. Furthermore, the maximum gain of the baroreflex curves was greater in HIH rats compared to control rats (Fig. 5). In addition, we determined chemoreflex function in both control and HIH rats. Chemorelfex was induced by intravenous injection of potassium cyanide (KCN, 40 µg/kg, 0.1 ml, iv bolus). After cyanide potassium injection, ABP and RSNA were increased, but HR was decreased in control group of rats. The changes of ABP, RSNA, and HR were significantly greater in HIH rats than in control rats (Fig 6).

## Discussion

This is the first study to investigate the mechanisms involved in the anti-depressor effect of HIH during acute hypoxia. We found that HIH attenuated the acute hypoxia-induced depressor response through enhancement of sympathetic outflow and increase in HR. It is likely that augmentation of baroreflex is responsible for the increases in HR and RSNA. Furthermore, opening of K_ATP_ channels may be involved in the anti-depressor effect of HIH. These data provide new insight into the mechanisms underlying anti-depressor effect of HIH.

The most interesting finding of this study is that acute hypoxia-induced depressor response was markedly reduced in HIH-treated rats, although HIH had little effect of the basal sympathetic tone. Acutely exposing to hypoxia leads to an increase in sympathetic outflow and a decrease in vagal activity [24], [27], [28], [29]. The sympathoexcitatory response is an important component to encounter the depressor response induced by acute hypoxia, which is caused by local vasodilation in response to a reduced blood oxygen concentration [16], [17]. Because the depressor and hypoxemia during acute hypoxia trigger both baroreflex and chemoreflex, in order to determine the role of these two reflexes in the responses of ABP, HR, and RSNA to acute hypoxia, we surgically removed bilateral carotid sinus and ascending depressor nerves, which mediate the baroreflex and chemcoreflex. Strikingly, after removal of bilateral carotid sinus and ascending depressor nerves, acute hypoxia-induced depressor, sympathoexcitatory, and tachycardiac responses were comparable in control and HIH rats. These data suggest that baroreflex and chemoreflex are involved in the enhanced sympathoexcitatory and tackycardia responses to acute hypoxia in HIH-treated rats.

The baroreflex and chemoreflex play important role in maintaining the homeostasis in ischemia and hypoxia. To determine the role of baroreflex in the enhanced sympahthoexcitatory response in HIH-treated rats, we directly evaluated baroreflex function through lowering and raising blood pressure with intravenous infusion of SNP and PE. We found that both reflexive RSNA and HR responses to PE-induced pressor and SNP-induced depressor were significantly greater in HIH-treated rats than in control group of rats. These data indicate that HIH treatment augments baroreflex function. Consistently, it has been shown that the HIH increases reduced baroreflex sensitivity in patients with chronic obstructive pulmonary disease (COPD) to the levels in healthy control subjects [12]. In support of this notion, we found that the same decreases in ABP triggered a greater increase in RSNA and HR in HIH-treated rats than in control rats, suggesting that the broreflex function was increased by HIH treatment. In addition, we determined chemoreflex function in rats received HIH. We found that the sympathoexcitatory response to intravenous injection of KCN was significantly greater in HIH rat than in control rats. These data provide direct evidence that HIH increases chemoreceptor sensitivity. Therefore, it is likely that increased baroreflex and chemoreflex function in HIH animal results in the augmented sympathoexcitory and tachycardia responses to acute hypoxia, which may be involved in the anti-depressor effect of HIH during acute hypoxia.

Although HIH treatment significantly increased sympathoexcitatory and tachycardia responses to acute hypoxia, the basal sympathetic vasomotor tone was not altered in HIH-treated rats. Our finding is consistent with previous studies. For example, 4-week HIH in young athletes does not significantly affect the steady-state cardiovascular hemodynamics (Fu et al. 2007). Furthermore, two consecutive night exposures to hypobaric hypoxia simulating an altitude of 4,000 m produces a small elevation of daytime blood pressure in healthy normotensive individuals, while the increased blood pressure returns to baseline at 90 min after the subject returned to basal altitude (Arabi et al. 1999). It is unlikely that mechanical ventilation may change the activation of chemoreceptors through affecting blood P_CO2_ level, because we maintained a stable arterial blood P_CO2_ level by adjusting the volume and frequency of mechanical ventilation during the experiment. Also, over-ventilation may cause activation of other mechanoreceptors in the lung tissue. The P_O2_ and P_CO2_ levels were maintained at stable levels, suggesting that the rats were unlikely over-ventilated and activated mechanoreceptors.

It is not clear about the precise mechanisms which were involved in the anti-depressor effect of HIH, although we found that HIH increased baroreflex and chemoreflex function. Nitric oxide (NO) plays an important in maintaining cardiovascular homeostasis and in the regulation of baroreflex and chemoreflex function [30], [31], [32], [33]. For example, it has been shown that blocking neuronal NO synthase with 7-nitroindazole alters baroreflex control of HR but not of RSNA in rabbits [31] However, we found that NO synthase inhibitor L-NAME failed to change the effect of HIH on RSNA, ABP and HR during acute hypoxia. The diverse effect of NO synthase inhibitor may be due to different agents, doses, and application methods used. Consistently, we found that administration of blocking NO synthase with L-NAME had no effect on the basal levels of ABP, RNSA, and HR. Thus, it seems that NO is not involved in the anti-depressor effect of HIH during acute hypoxia.

It has been shown that cromakalin, an opener of K_ATP_ channels, facilitates the carotid baroreflex through increasing stretch of baroreceptor by dilation of sinus vessel wall (Yin et al. 2000). Therefore, we determine the role of K_ATP_ in the anti-depressor effect of HIH during acute hypoxia. Strikingly, blocking K_ATP_ channels with its selective antagonist glibenclamide abolished the difference between acute hypoxia-induced depressor and sympathoexcitatory responses in HIH and control rats. These data suggest that K_ATP_ channel is importantly involved in the attenuated depressor and increased sympathoexcitory and tachycardia responses in HIH rats. However, we found that glibenclamide had little effect on acute hypoxia-induced depressor and sympathoexcitatory responses in control rats, suggesting that K_ATP_ channels are not involved in decrease in blood pressure caused by acute hypoxia in control rats. It has been shown that K_ATP_ channels are widely expressed in excitable cells and play critical role in the regulation of cell excitability [34]. In this study, we blocked K_ATP_ channels systemically; it is thus not clear what is the exact sites glibenclamide act on. Activation of K_ATP_ channels, which are distributed in vascular smooth muscle, leads to hyperpolarization and relaxation of vascular smooth muscle [35], [36]. However, K_ATP_ channels blocker glibenclamide had little effect on the basal blood pressure and sympathetic activity in both control and HIH-treated rats. It has been shown that K_ATP_ channels express on the components along the pathways of baroreflex. For example, K_ATP_ channel-forming units, Kir6.2, and its regulatory units, sulfonylurea receptor (SUR) are abundant in rat nodose ganglia, which is an important component in baroreflex pathway [37], [38]. Furthermore, K_ATP_ channels are detected in the brainstem nucleus such as nucleus solitary tract, rostro-ventrolateral medulla, and caudal ventrolateral medulla, which are involved in baroreflex [39], [40], [41]. Together with data showing that HIH treatment increased the sensitivity of baroreflex, it is possible that HIH induces activation of K_ATP_ channels in the neurons which are involved in baroreflex. Therefore, the underlying mechanisms warrantee future studies.

## Materials and Methods

### Hypobaric Intermittent Hypoxia

The animal experimental protocols employed in this study were approved by the Committee on the Use of Animals for Teaching and Research of Hebei Medical University. These protocols also conformed to the Guide for the Care and Use of Laboratory Animals, published by the National Institutes of Health (NIH Publication No. 85-23, revised 1996). Male Sprague-Dawley rats (200∼240 g) were used in this study. These rats were obtained from the Experimental Animal Center of HeBei Province and were randomly divided into four groups: Control group, HIH treatment for 14-day (HIH-14), 28-day (HIH-28), and 42-day (HIH-42). All the experiments were conducted in compliance with the Guide for the Care and Use of Laboratory Animals (National Research Council, 1996). The HIH rats were exposed to simulated high-altitude hypoxia in a hypobaric chamber mimicking 5000 m altitude (O_2_∶11.1%) 6 h per day for 14 d, 28 d, and 42 d, respectively. The pressure (406±5 mmHg) inside the hypobaric chamber was monitored and maintained by a vacuum pump at room temperature (23–25°C). The control animals breathed normal room air. All of the rats have free access to regular food and water. The body weight of rats was recorded each week for the whole experimental period. Arterial blood pressure (ABP) of these rats was measured conscious condition by using tail-cuff method weekly (AD Instrument, Australia). The animals were placed inside a warm black cuff (about 25°C) for 10 min to calm the animals and dilate the tail blood vessels before ABP measurement. The ABP was measured between 5∶00 pm and 7∶00 pm.

### Recording of ABP, HR, and Renal Sympathetic Nerve Activity (RSNA) in Anesthetized Rats

The rats were anesthetized with 25% urethane (1.25 g/kg, i.p) and additional doses were added when required. We recognize that anesthetics may have confounding effect on autonomic nervous activity and blood pressure. It has been shown that urethane has a minimal effect on sympathetic vasomotor tone and baroreflex than other anesthetics such as pentobarbital sodium [42]. Adequate depth of anesthesia was verified by the absence of responses to noxious pinch of the paw. Supplemental doses of urethane (50–100 mg/kg) were administered to maintain an appropriate level of anesthesia. The trachea was cannulated and the rats were ventilated mechanically (Harvard Apparatus Inc, Holliston, MA, USA). The arterial O_2_ and CO_2_ pressure (P_O2_ and P_CO2_, respectively) were determined in blood samples withdrawn from left carotid artery. P_O2_ and P_CO2_ were maintained at the same levels prior to cannulation by adjusting the volume and frequency of mechanical ventilation. The right femoral vein was cannulated for drug administration and blood sample withdraw, which was used for measuring venous blood gas (Model Medica Easy Blood Gas, USA). Arterial and vein blood gases were measured by using blood samples withdrawn from femoral vein and artery, respectively. The right femoral artery was also cannulated for recording ABP. The HR was calculated from ABP signal. Rectal temperature was monitored and kept at about 37°C with a servo-controlled heat blanket. At the end of the experiments, rats were killed by overdose urethane (iv).

The left kidney was exposed via a retroperitoneal approach. A branch of renal sympathetic nerve was carefully isolated from the surrounding tissue and clamped distally to eliminate the afferent activity. The renal sympathetic nerve was hooked onto a bipolar sliver electrode for recording and immediately immersed in warm liquid paraffin (37°C) to avoid nerve dry and interference. RSNA was filtered (160 Hz∼1000 Hz) and amplified via a set of biological function experimental system (RM-6240 Chengdu Instrument Factory, China). The amplified signal was integrated (160 ms) and acquired with computer software. At the end of the experiment, the proximal end of the nerve was clamped to get the noise level of RSNA for reference and the data was expressed as percent change from control value. The baseline level of RSNA was defined as 100% and the deviation induced by acute hypoxia were expressed as a percentage of the baseline [43], [44]. Acute hypoxia was induced by delivering mixed gas containing 10% O_2_ and 90%N_2_ through the trachea cannula for 2 min. In some experiments, the baseline level of RSNA, measured at the highest blood pressure induced by phenylephrine, was expressed as 0. The baseline level of RSNA, measured at the lowest blood pressure induced by sodium nitroprusside, was expressed as 100. Deviations from this baseline during PE and SNP infusions were normalized between the 0 and 100% levels of RSNA. The bilateral aortic depressor nerves, carotid sinus nerves and recurrent laryngeal nerves were isolated and sectioned according to protocols. Completeness of baro-denervaion was ensured by the absence of HR decreasing during hypertension induced by PE (8 µg/kg, iv, 0.1 ml bolus).

### Drugs

Potassium cyanide (KCN), phenylephrine (PE), sodium nitroprusside (SNP), NG-Nitro-L-arginine methyl ester (L-NAME), and glibenclamide were purchased from Sigma. Glibenclamide was dissolved in dimethylsulphoxide. No change was observed during intravenous injection of the final concentration of DMSO (0.1%). Other drugs were dissolved in saline and prepared just before the experiments.

### Data Analysis

During PE and SNP infusions, RSNA and ABP were averaged over sustained changes in mean ABP. Sigmoid logistic curves were approximated to experimental data points (15 points per curve) for each animal, according to the model described by Kent et al [45], with the use of Origin 6.0 Professional software for Windows and the formula: RSNA  = A/{1+ exp[B(ABP - C)]}+D, where *Y* is RSNA or HR, *A* is the RSNA or HR range, *B* is the slope coefficient, *C* is the MAP at the midpoint of the RSNA or HR range (which is also the point of maximum gain), and *D* is the minimum RSNA or HR [45], [46], [47]. The baroreflex curves were differentiated to determine the gain of the HR and RSNA components of the baroreflex across the MAP range [45], [46], [47]. The maximum gain was determined by taking the first derivative of the baroreflex curve described by the logistic equation. Baroreflex response curves were constructed, and their parameters were calculated for each nerve in each animal and then averaged across animals [43], [44], [45], [48], [49]. All values were expressed as mean ± SEM. The significance of differences within groups was determined by paired t-test. The significance of differences between groups were determined by one-way analysis of variance (ANOVA) and further analyzed by Student-Newman-Keuls test. Differences were considered statistically when P<0.05.
