# Correction: Hypobaric Intermittent Hypoxia Attenuates Hypoxia-induced Depressor Response

**DOI:** 10.1371/annotation/1b9eb18d-b23f-4a5c-8357-e517e209e821

**Published:** 2013-04-12

**Authors:** Fang Cui, Lu Gao, Fang Yuan, Ze-Fei Dong, Zhao-Nian Zhou, David D. Kline, Yi Zhang, De-Pei Li

In Figure 6, Panel D, the delta HR (y-axis) number should be as follows: -25, -50, -75, -100. 

